# Isolation and Characterization of a Novel Recombinant Classical Pseudorabies Virus in the Context of the Variant Strains Pandemic in China

**DOI:** 10.3390/v15091966

**Published:** 2023-09-20

**Authors:** Zhengmin Lian, Panrao Liu, Zhenbang Zhu, Zhe Sun, Xiuling Yu, Junhua Deng, Ruichao Li, Xiangdong Li, Kegong Tian

**Affiliations:** 1 Jiangsu Co-Innovation Center for Prevention and Control of Important Animal Infectious Diseases and Zoonoses, College of Veterinary Medicine, Yangzhou University, Yangzhou 225009, China; lian_zm@126.com (Z.L.); 007617@yzu.edu.cn (P.L.);; 2 Luoyang Putai Biotech Co., Ltd., Luoyang 471003, China

**Keywords:** pseudorabies virus, classical strain, phylogenetic analysis, recombination, pathogenicity

## Abstract

Pseudorabies virus (PRV) variants were discovered in immunized pigs in Northern China and have become the dominant strains since 2011, which caused huge economic losses. In this study, a classical PRV strain was successfully isolated in a PRV gE positive swine farm. The complete genome sequence was obtained using a high-throughput sequencing method and the virus was named JS-2020. The nucleotide homology analysis and phylogenetic tree based on complete genome sequences or gC gene showed that the JS-2020 strain was relatively close to the classical Ea strain in genotype II clade. However, a large number of amino acid variations occurred in the JS-2020 strain compared with the Ea strain, including multiple immunogenic and virulence-related genes. In particular, the gE protein of JS-2020 was similar to earlier Chinese PRV strains without Aspartate insertion. However, the amino acid variations analysis based on major immunogenic and virulence-related genes showed that the JS-2020 strain was not only homologous with earlier PRV strains, but also with strains isolated in recent years. Moreover, the JS-2020 strain was identified as a recombinant between the GXGG-2016 and HLJ-2013 strains. The pathogenicity analysis proved that the PRV JS-2020 strain has typical neurogenic infections and a strong pathogenicity in mice. Together, a novel recombinant classical strain was isolated and characterized in the context of the PRV variant pandemic in China. This study provided some valuable information for the study of the evolution of PRV in China.

## 1. Introduction

Pseudorabies virus (PRV) is a highly pathogenic and infectious pathogen in pigs, which causes pseudorabies (PR) or Aujeszky’s disease (AD) and is characterized by neurological symptoms, fever and itchiness. AD leads to abortion and stillbirth in swine, growth retardation in growing pigs, and high mortality in piglets, resulting in huge economic losses in pig production. PRV is a double-stranded linear DNA virus belonging to the Herpesviridae family, Alphaherpesvirinae subfamily and Varicellovirus genus. The genomes of herpesvirus were divided into six classes (A to F). The PRV genome belongs to the D class, which is similar to the varicella-zoster virus (VZV) genome [1]. The first complete DNA sequence of the PRV genome was obtained from several published incomplete sequences and multiple newly sequenced fragments derived from different strains [2]. The first full genome characterization of a single PRV strain (Bartha strain) was revealed using Illumina high-throughput sequencing [3]. These studies showed that PRV was organized into a unique long (UL) region, a unique short (US) region and two large inverted and terminal repeats (IR, TR) flanking the US region.

The earliest sporadic outbreaks of PRV were reported in the United States in 1813 and then PRV was spread around the world [1]. With the widespread use of the PRV Bartha-K61 strain, an attenuated live vaccine, PRV was effectively controlled in pigs [4]. Furthermore, it has been eradicated in the United States, Netherlands and various European countries [5]. In China, PRV was discovered in 1950s. With the use of the commercial Bartha-K61 vaccine since the 1970s, PRV was effectively controlled in Chinese swine farms. However, since 2011, the PRV variant strain was discovered in pigs immunized with the Bartha-K61 vaccine in Northern China, and then spread rapidly almost nationwide [6,7]. The epidemiological investigation revealed that the positive rate of wild-type PRV was 8.27% between 2012 and 2017, and it even reached 12% between 2012 and 2013 [8]. Although the Bartha-K61 vaccine was widely used in swine farms, more and more outbreaks of PR were reported in vaccinated swine farms since the variant strain was discovered [9,10,11]. Several research studies have shown that the Bartha-K61 vaccine could not provide full protection against the PRV variant strains [7,12,13]. Compared with classical PRV strains, the PRV variant showed stronger pathogenicity in pigs [10,14]. In recent years, several novel vaccines based on PRV variant strains have been developed and evaluated, including the gE/gI-deleted inactivated vaccine based on the PRV ZJ01 strain [15], the inactivated vaccine (gE-deleted) and the live attenuated vaccine (gE/gI/TK-deleted) based on the PRV HN1201 strain [16,17].

With the wide application of high-throughput sequencing technology, multiple complete genome sequences of PRV variant strains were reported and characterized. However, the novel classical strains were less reported on. In this study, a novel classical PRV strain was isolated in the context of the PRV variant pandemic in China. Its complete genome sequence was obtained using Illumina high-throughput sequencing. The genome characteristics, genetic evolution and amino acid variations were analyzed. Furthermore, the biological characteristics and pathogenicity were further revealed and tested.

## 2. Materials and Methods

### 2.1. Cells and Virus Infection

PK-15 cells were purchased from the American Type Culture Collection (ATCC) and cultured in Dulbecco’s modified Eagle medium (DMEM) (Solarbio, Shanghai, China) supplemented with 10% fetal bovine serum (FBS, Thermo Fisher Scientific, Waltham, MA, USA) at 37 °C in 5% CO_2_. Clinical brain tissue samples were collected from a case of PR-suspected aborted swine in Jiangsu Province of China in 2020. The fever and loss of appetite were observed from aborted swine. It was a PRV negative farm, and the pigs were not vaccinated with any PRV vaccines in recent years. Samples were homogenized and the PK-15 cells were infected with filtered supernatant for 1 h. Next, the infected cells were washed with phosphate-buffered saline (PBS) and incubated in DMEM supplemented with 1% FBS.

### 2.2. PCR Identification and Growth Curves

The supernatant samples from infected PK-15 cells which showed PRV-like cytopathic effects (CPEs), were purified using three rounds of plaque purification to obtain purified PRV. Total DNA strands of purified PRV samples were extracted using viral genomic DNA extraction kit according to the manufacturer’s instructions (TIANGEN, China). Next, purified DNA samples were identified with polymerase chain reaction (PCR) using PRV specific primers pairs for gD gene (F: 5′ CAG GAG GAC GAG CTG GGG CT -3′ and R: 5′ GTC CAC GCC CCG CTT GAA GCT -3′).

The purified PRV JS-2020 and Bartha strain were inoculated with PK-15 cells at 0.1 MOI in DMEM supplemented with 1% FBS, and the supernatants were harvested at 2, 6, 12, 24, 36 and 48 h post infection. The viral titers of supernatants were tested using PK-15 cells.

### 2.3. Immunofluorescence

PK-15 cells were infected with JS-2020 at 0.1 MOI in DMEM and supplemented with 1% FBS; cell samples were collected at 24 h post infection. For immunofluorescence, the infected PK-15 cells were fixed with 4% paraformaldehyde for 10 min and permeabilized with 0.5% Triton X-100 at room temperature. Cells were blocked with 3% bovine serum albumin (BSA) for 1 h at room temperature and incubated with PRV gB protein antibody (A general gift from Prof. Beibei Chu at Henan Agricultura University) overnight at 4 °C. Following three washes with PBS, cells were incubated with an Alexa fluor 555-conjugated anti-mouse IgG (Cell Signaling Technology, Danvers, MA, USA) for 1 h at the room temperature. The cells were visualized with an inverted fluorescence microscope (U-HGLGPS, OLYMPUS, Tokyo, Japan).

### 2.4. Complete Genome Sequencing and Analysis

Purified genomic DNA of PRV JS-2020 strain was sequenced through next-generation sequencing (NGS) technology using Illumina paired-end sequencing (Sangon Biotech, Shanghai, China). The complete genome sequence was annotated using SnapGene 6.0 software and submitted to the GenBank database (GenBank accession number: OR271601).

The complete sequences of JS-2020 strain were aligned with other 15 PRV strains (Table 1), the nucleotide homology analysis was performed using MegAlign module of DNASTAR Lasergene 7 software. Phylogenetic trees of genomic and gC sequences were constructed using maximum likelihood (ML) method of MEGA11 software (V11.0.13).

### 2.5. Amino Acid Variations Analysis

Amino acid sequence alignment of JS-2020 ORFs were performed using BLAST on the NCBI website (https://blast.ncbi.nlm.nih.gov/Blast.cgi?PROGRAM=blastp&PAGE_TYPE=BlastSearch&LINK_LOC=blasthome, accessed on 20 June 2023). The amino acid sequence homology and phylogenetic trees of PRV major immunogenic and virulence-related genes (including gB, gC, gD, gE, gI and TK) were analyzed using maximum likelihood (ML) method with MEGA11 software (V11.0.13).

### 2.6. Recombination Analysis

RDP4 software (V4.101) was used to detect the potential recombination signals in JS-2020 strain with Bootscan, 3seq, PhylPro, Maxchi, SiScan and Chimaera algorithms. Then, the major recombination events were further validated with SimPlot 3.5.1 software with a sliding window of 2000 nucleotides which moved every 200 nucleotide steps.

### 2.7. Animal Experiments

The purified PRV JS-2020 strain was diluted to 10^4.5^ TCID_50_/mL in DMED. Six-week-old specific pathogen-free (SPF) BALB/c mice (Comparative Medicine Center of Yangzhou University) were randomly divided into two groups including DMEM group and PRV infected group. The 5 mice of PRV infected group were infected with 10^3.5^ TCID_50_ PRV JS-2020 strain in 100 μL DMED by injecting intraperitoneally. Another 5 mice of DMEM group were intraperitoneally injected with 100 μL DMED. Mice were monitored daily, and the survival rates were recorded for 7 days.

### 2.8. Histopathology and Immunohistochemistry Staining

Brain tissues samples were collected from dead mice of PRV JS-2020 strain infected group and surviving mice of DMEM group. Samples were fixed with 10% formaldehyde, placed into paraffin blocks, and cut into sections. The hematoxylin and eosin staining were applied to sections for histopathological examination. PRV specific antibody (anti-gB protein) was used for immunohistochemistry staining as described previously [18].

## 3. Results

### 3.1. Isolation and Identification of PRV JS-2020 Strain

**Figure 1:**
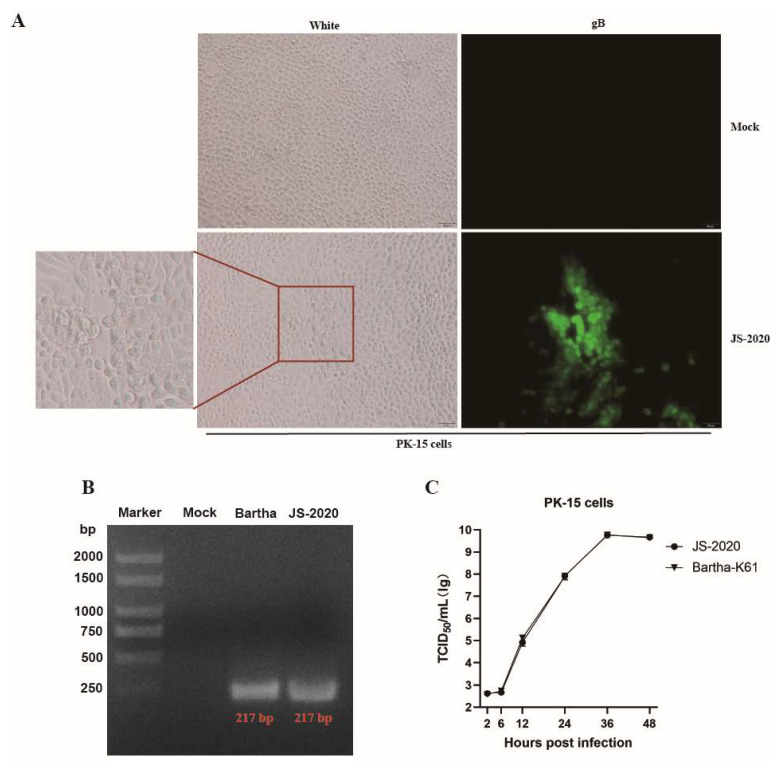
Isolation and identification of PRV JS-2020 strain. (**A**) PRV-like CPEs were observed within 24 h post infection, and the gB protein of PRV was positive (IFA). (**B**) PCR identification of purified JS-2020 strain (gD gene, where Bartha was used as a positive control). (**C**) Growth curves of JS-2020 strain.

The PRV gE positive brain tissue samples from aborted piglets were identified with the real-time PCR method. Supernatants of the PRV gE-positive tissues were incubated in PK-15 cells and typical CPEs of PRV were observed within 24 h post infection (hpi) (Figure 1A). Moreover, the results of IFA showed that PRV gB protein was detected in infected PK-15 cells (Figure 1A). After three rounds of plaque purification and PCR identification (Figure 1B), the purified PRV was obtained and named JS-2020. Results of growth curves showed the highly efficient replication capability of JS-2020 in PK-15 cells (Figure 1C). The viral titers of JS-2020 in infected cells’ supernatants increased rapidly from 6 hpi to 12 hpi, and peaked with a viral copy number of more than 10^9^ TCID_50_/mL after 36 hpi (Figure 1C). This result was similar to the Bartha-K61’s outcome. These results indicated that a field strain of PRV was successfully isolated from the clinical samples and grew well in PK-15 cells.

### 3.2. Genomic Characterization of the JS-2020 Strain

**Figure 2:**
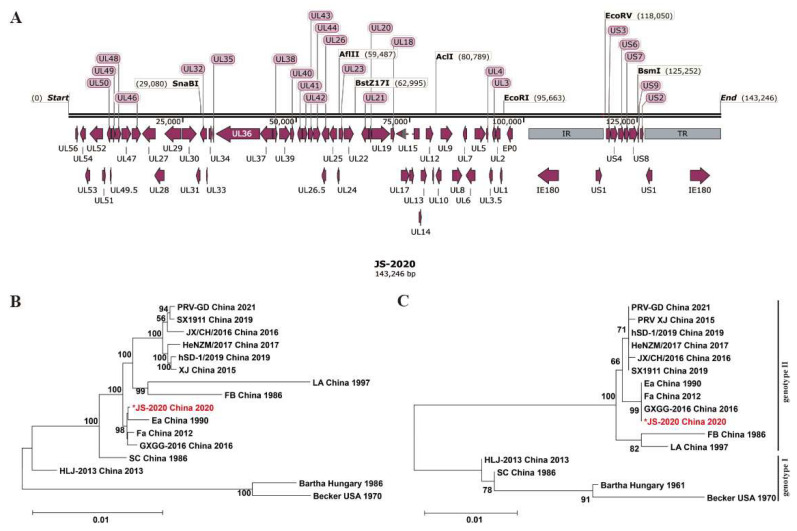
Genomic characterization of PRV JS-2020 strain. Complete genome distribution of JS-2020 strain (**A**) and the phylogenetic trees based on both complete sequences (**B**) and gC (**C**). “*” indicates the target PRV strain in this study.

To identify the genetic characteristics of the JS-2020 strain, the complete genome sequences were obtained using the high-throughput sequencing method. The complete genome length of the JS-2020 strain was 143,246 bp, which encodes 69 open reading frames (ORFs) (Figure 2A). The genome sequence was divided into the following four parts: UL (101,287 bp), US (9183 bp), IR (16,388 bp) and TR (16,388 bp) (Table 2). GC content was 74%, which was similar to other published PRV strains [4].

Based on complete genome sequences, the nucleotide homology analysis revealed that JS-2020 shared 91.3–99% homology with other PRV strains. In addition, it had the highest homology with the Ea strain (99.8%), which is a Chinese classical PRV strain isolated in 1990 (Table 3). Moreover, the phylogenetic trees based on both genomic and gC sequences were constructed and analyzed (Figure 2B,C). The phylogenetic tree based on gC showed that the PRV strains were classified into genotype I and genotype II, and the JS-2020 strain was clustered within genotype II. The results based on the complete genome showed that the JS-2020 strain also clustered with Chinese PRV strains and had the closest genetic evolutionary relationship with the Ea strain, which was a Chinese classical PRV strain isolated in 1990. These results, which were based on the analysis of genome sequences, suggested that a classical PRV strain was isolated in the context of the PRV variant pandemic in China.

### 3.3. Amino Acid Variations Analysis of the JS-2020 Strain

Compared with the Ea strain, a total of 27 proteins of the JS-2020 strain were different, containing 86 mutations, 7 deletions and 20 insertions (Table 4). The major amino acid variations occurred in UL47 (7 aa), UL27 (6 aa), UL36 (34 aa), IE180 (7 aa), US1 (21 aa) and US3 (5 aa).

**Figure 3:**
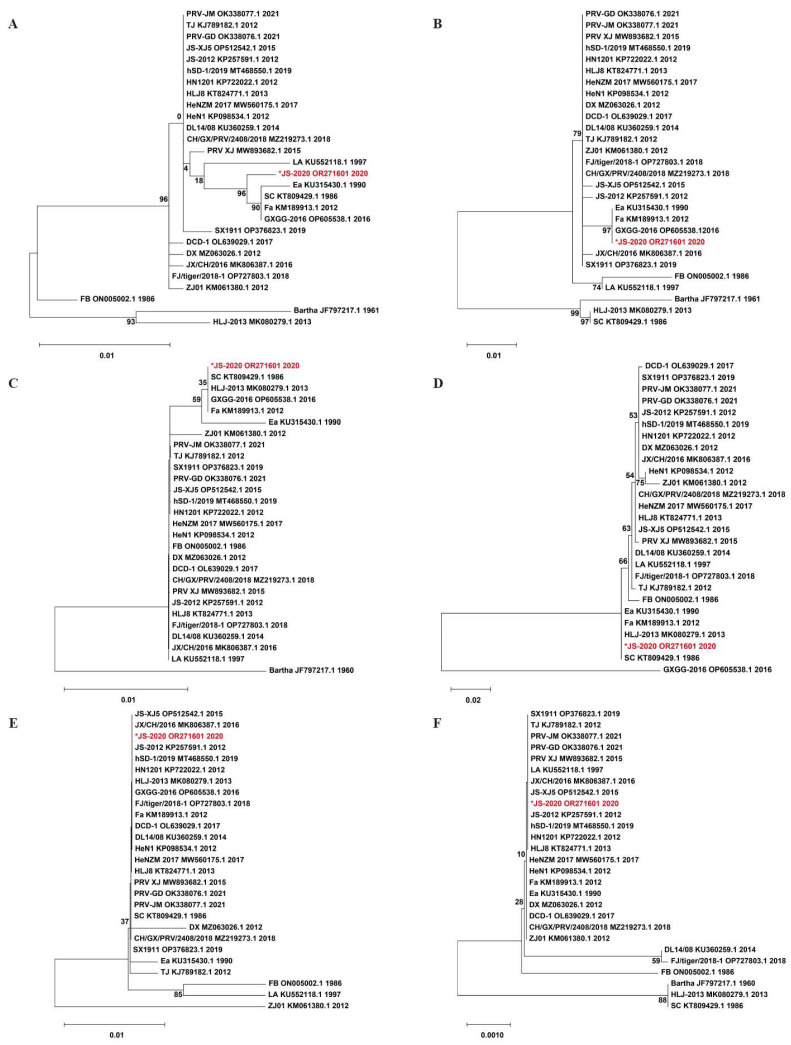
Phylogenetic trees that are based on amino acid sequences of gB (**A**), gC (**B**), gD (**C**), gE (**D**), gI (**E**) and TK (**F**). “*” indicates the PRV isolate in this study.

To assess the variations of major immunogenic and virulence-related genes (including gB, gC, gD, gE, gI and TK), the amino acid sequence of the JS-2020 strain was compared with 24 Chinese PRV strains and the Bartha strain. The homology analysis results showed that the gI and TK of JS-2020 was highly conserved (sharing 100% homology with most of the Chinese PRV strains) and gB, gC, gD and gE shared lower homology with other PRV strains (gB 96.5–99.7%, gC 92.4–100%, gD 97.3–100% and gE 83.9–100%). Moreover, the phylogenetic trees based on amino acid sequence of gB, gC, gD, gE, gI and TK were constructed and analyzed (Figure 3). The gB protein of the JS-2020 strain had the highest homology with the Fa, GXGG-2016 and SC strain (99.7%), but their evolutionary relationships belong to different branches (Figure 3A). The gC protein has 100% homology and the closest evolutionary relationship with the Ea, Fa and GXGG-2016 strain (Figure 3B). The gD protein has the highest homology (100%) and belongs to same branch of evolutionary relationship with the Fa, GXGG-2016, HLJ-2013 and SC strain. (Figure 3C). The gE protein has the highest homology (100%) and the closest evolutionary relationship with Ea, Fa, HLJ-2013 and SC strain (Figure 3D). The evolutionary relationships of highly conserved gI and TK proteins are similar to homology analysis results, which are also similar to most of the Chinese PRV strains (Figure 3E,F).

**Figure 4:**
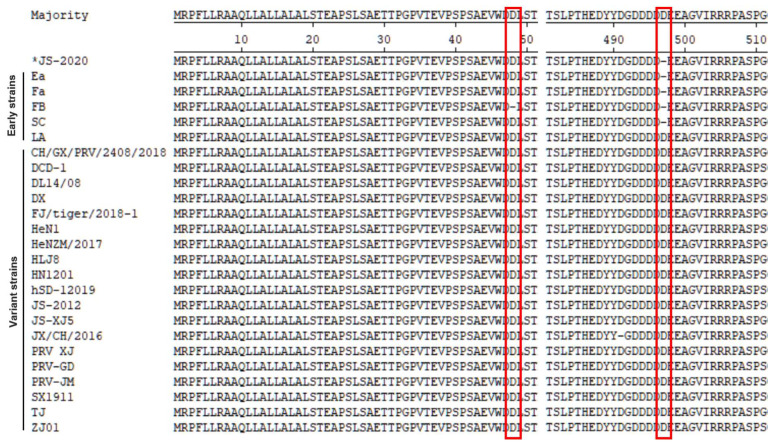
Alignment of amino acid sequences of PRV gE protein. JS-2020 strain is similar to earlier Chinese PRV strains without Asp insertion at amino acid position 497. Red lines indicate two Asp insertions in the variant strains.

The results of previous studies showed that the gE protein of the PRV variant strain contained two Aspartate (Asp, D) insertions when compared with earlier PRV strains that were isolated from China. Although Asp insertions were also observed in a few early PRV strains, the insertions in the variant strains were highly conserved [7,9]. The results of the amino acid sequence analysis showed that the gE protein of the JS-2020 strain is similar to the earlier Chinese PRV strains without the Asp insertion at the amino acid position 497 (Figure 4). These results further proved that a classical PRV strain was isolated, which is similar to earlier Chinese PRV strains.

### 3.4. Recombination Analysis

**Figure 5:**
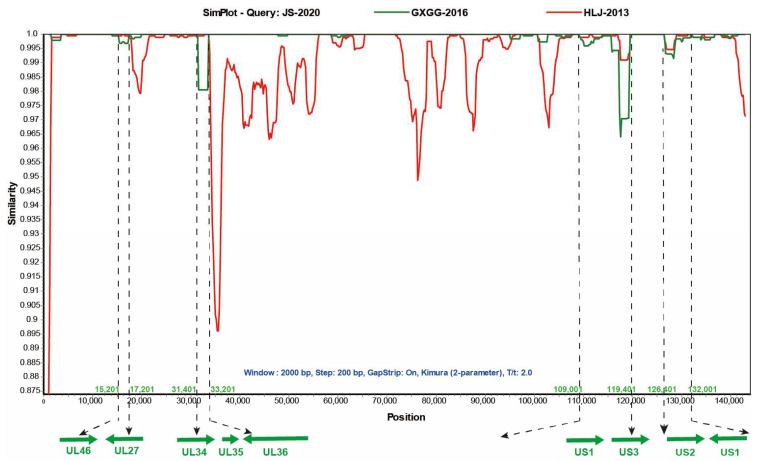
Recombination analysis of JS-2020 strain using Simplot 3.5.1 software. The four recombination regions are shown with dotted lines. Parameters: Window 2000 bp, Step 200 bp, GapStrip On, Kimura (2-parameter), T/t 2.0.

Multiple studies have shown that recombinant events were found in a few isolated Chinese PRV strains [9,19,20]. The homology analysis of the JS-2020 results showed that partial genes of the JS-2020 have high homology and close evolutionary relationship not only with earlier PRV strain (Ea), but also with recent strains (GXGG-2016). These results suggested that the JS-2020 might be a recombinant strain. To test whether there are any recombination signals in the JS-2020 with other Chinese PRV strains (as shown in Table 5) and the Bartha strain, recombinant analysis was performed using RDP4 software (V4.101). The results showed that several major recombination events were detected in the JS-2020 strain with Bootscan, 3seq, PhylPro, Maxchi, SiScan and Chimaera algorithms. In addition, the major backbone of the JS-2020 was the GXGG-2016 strain, and the major recombination regions were obtained from the HLJ-2013 strain (minor backbone). There were no Ea and Fa strains associated with the recombination signals in the JS-2020 strain, although they shared high homology. Moreover, the recombination events were further verified using Simplot 3.5.1 software. The result showed that four potential major recombination regions which form the HLJ-2013 strain were detected and that they were located at 15,201 to 17201; 31,401 to 33,201; 109,401 to 119,401 and 126,401 to 132,001 (Figure 5). The major recombination regions include partial UL46, UL27, UL34, UL36 and US3 ORFs and complete UL35, US1 and US2 ORFs (Figure 5). The other regions of the JS-2020 strain were highly similar to the GXGG-2016 strain. These results indicated that the JS-2020 and the GXGG-2016 strain share a common parental strain and most of the fragments in the JS-2020 strain come from the GXGG-2016 strain. However, there is a continuous deletion of 69 amino acids in the TK gene of the GXGG-2016 strain [20], but the JS-2020 does not undergo deletion. Therefore, this indicates that the TK gene of the JS-2020 comes from other PRV strains.

### 3.5. Pathogenicity Analysis

**Figure 6:**
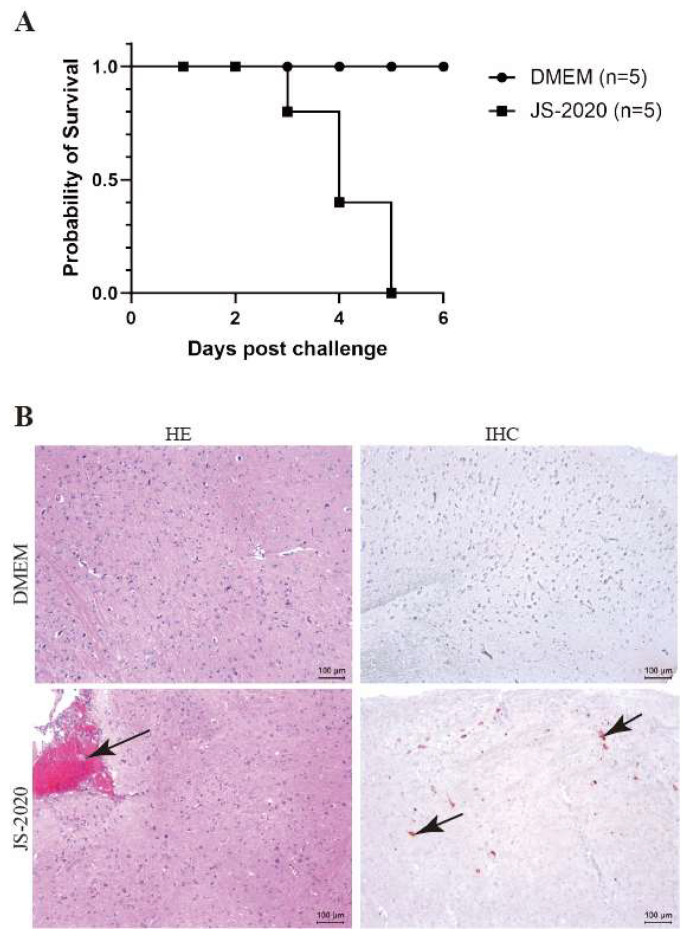
Pathogenicity of JS-2020 strain in mice. (**A**) Six-week-old SPF BALB/c mice were infected with 10^3.5^ TCID50 PRV JS-2020, survival of each group was recorded and the survival curves were generated. (**B**) The histopathological examination (HE) and immunohistochemistry staining (IHC) of brain tissues.

Mice are commonly used as animal models since they show neurogenic infections of the central nervous systems (CNS) with high mortalities in a productive PRV infection [21,22]. To assess the pathogenicity of the JS-2020 strain, the six-week-old specific pathogen-free (SPF) BALB/c mice were infected with the PRV JS-2020 strain or the DMEM by injecting, intraperitoneally. The results showed that the mice infected with the PRV JS-2020 strain began to die on the third day post infection (Figure 6A). All PRV infected mice died on firth day post infection and the symptoms associated with the PRV infection (nervous symptoms and dead mice) were not observed in the DMEM group. In our previous study results, the higher dose of PRV Bartha -K61 (50 μL 1 × 10^5^ TCID_50_/mL) infected mice started to die as early as the fifth day post infection. Therefore, the JS-2020 showed higher pathogenicity than Bartha-K61 in mice [23].

Next, histopathological examination and immunohistochemistry staining of brain tissues were performed to evaluate the neurovirulence of the JS-2020 strain. The hematoxylin-eosin (HE) staining results showed that the meningeal congestion was observed in mice infected with the PRV JS-2020 strain (Figure 6B, HE). Immunohistochemistry staining results revealed that PRV antigens were positive in brain tissues of the mice infected with the PRV JS-2020 strain (Figure 6B, IHC). No lesions or PRV antigens were observed in the brain tissues of mice in the DMEM group. These results indicated that the PRV JS-2020 strain has typical neurogenic infections and a strong pathogenicity in mice.

## 4. Discussion

Since the PRV variant strains were discovered in 2011 in China, it has spread to most areas of China and caused huge economic losses. Next, more and more PRV variant strains were isolated and identified. To control the spread of newly emerged PRV variants, several effective vaccines based on PRV variants have been developed and applied in recent years in China. However, PRV exhibits a definite neurotropism and results in acute infection in piglets or in the establishment of latent infection in trigeminal ganglion neuros [1,21]. Based on its characteristic of latent infection, the earlier PRV strains were able to maintain long-term infection in swine farms. These PRV strains can be reactivated and spread to healthy pigs. In this study, a classical PRV strain (JS-2020) was isolated and identified from PRV-infected pigs in 2020. It proved that the earlier PRV strains still persist in Chinese swine farms in the context of the PRV variant pandemic.

The complete genomic sequences of the PRV JS-2020 strain were obtained using a high-throughput sequencing method. It had similar genome organization, including a unique long (UL) region, a unique short (US) region and two inverted repeats (IR and TR) along with other published PRV strains. Based on complete genome sequences, the JS-2020 strain has high homology and a close evolutionary relationship with the Ea strain (an earlier classical PRV strain isolated in 1990 in China). In addition, it was classified into genotype II with most of the other Chinese PRV strains based on the gC phylogenetic tree. Compared with the Ea strain, a large number of amino acid variations occurred in the JS-2020 strain, including multiple immunogenic and virulence-related genes.

It was reported that the gB, gC, gD, gE, gI and TK genes are major immunogenic and virulence-related genes of PRV. The gB protein is one of envelope glycoproteins and major viral antigen in PRV, participates in the processes of viral entry and cell-to-cell spread [24,25,26]. The gC protein was regarded as a viral adhesion and immune response related glycoprotein [27]. The cell receptor nectin-1, an immunoglobulin-like cell adhesion molecule, is engaged by the gD protein at an early step of PRV infection. It was reported that the special antibody of gD protein was able to block PRV attachment to cell [28,29]. Both gE and gI are neurovirulence associated proteins of PRV. Deletion of gE or gI severely reduces anterograde spread capacity of PRV in processes of axonal transport [30,31]. The TK gene codes thymidine kinase of PRV. The pathogenicity of TK mutant PRV was highly attenuated in mice, rabbits and pigs [1]. The amino acid variations analysis based on these immunogenic and virulence-related genes showed that the gB, gC, gD and gE genes of the JS-2020 strain were not only homologous with earlier PRV strains (the Ea, Fa or SC strains), but also with strains isolated in recent years (the GXGG-2016 or HLJ-2013 strains). In particular, the gE gene of the JS-2020 strain was most similar to the earlier strains without an Asp insertion when compared to those with variant strains isolated in recent years. Both the gI and TK gene of the JS-2020 strain were highly conserved compared with most of the Chinese PRV strains.

It was reported that the GXGG-2016 and HLJ-2013 strains were classical PRV strains, and they were also homologous with earlier Chinese strains from previous studies [20,32]. The analysis of homology and variations based on nucleotide or amino acid sequences suggested that the JS-2020 strain might have evolved from the GXGG-2016 or HLJ-2013 strain. In recent studies, multiple recombinant PRV strains were identified, such as the JSY13, SC and FJ strains. The recombination regions of the JSY13 and SC strains were regarded as a Bartha strain [9,19]. The FJ strain was regarded as a recombination strain between the HLJ8 and Ea strains [33]. The evolutionary and recombination analysis based on a large number of PRV complete genome sequences indicated that novel PRV variants might evolve from classical PRV strains through recombination mechanisms [34]. In this study, the recombination events were further predicted using related software. The JS-2020 strain was identified as a recombinant, its major sequences was highly similar to GXGG-2016 strain and a fraction of recombination regions were from the HLJ-2013 strain. In summary, a recombinant classical PRV strain was isolated and characterized in this study. These results will provide some evidence for the PRV-evolution-related studies. Moreover, it indicated that the classical PRV strains were still spreading among Chinese swine farms in the context of the PRV variant pandemic and that the novel natural recombinant virus is constantly being produced.

## Figures and Tables

**Table 1:** Information of pseudorabies virus strains for gC gene and complete genome analysis.

Strain	Accession Number	Country	Isolation Date
HeNZM/2017	MW560175.1	China	2017
JX/CH/2016	MK806387.1	China	2016
LA	KU552118.1	China	1997
Ea	KU315430.1	China	1990
Fa	KM189913.1	China	2012
FB	ON005002.1	China	1986
hSD-1/2019	MT468550.1	China	2019
PRV XJ	MW893682.1	China	2015
PRV-GD	OK338076.1	China	2021
SX1911	OP376823.1	China	2019
Becker	JF797219.1	USA	1970
SC	KT809429.1	China	1986
Bartha	JF797217.1	Hungary	1961
GXGG-2016	OP605538.1	China	2016
HLJ-2013	MK080279.1	China	2013
JS-2020	OR271601	China	2020

**Table 2:** Genome organization and location of PRV JS-2020 strain.

Region	Start (5′)	End (3′)	Length (bp)
UL	1	101,287	101,287
IR	101,288	117,675	16,388
US	117,676	126,858	9183
TR	126,858	143,246	16,388

**Table 3:** Nucleotide homology of 13 PRV strains compared with JS-2020 strain.

		Nucleotide Homology % (Complete Genome)													
PRV Strain	JX/CH/2016	LA	Ea	Fa	FB	hSD-1/2019	PRV XJ	PRV-GD	SX1911	Becke	SC	Bartha	HLJ-2013	GXGG-2016	JS-2020
HeNZM/2017	97.1	94.1	96.4	96.1	94.6	96.6	96.9	95.6	97.2	91.7	95.7	90.3	95.5	96.3	96.8
JX/CH/2016		94.7	97.1	97	95.4	97.6	98.4	95.6	97.8	91.9	96.7	90.9	96.5	97.1	97.8
LA			94.9	94.8	93.5	94.3	94.7	93.3	94.3	92.1	94.2	90.6	94.7	94.7	95.1
Ea				98.3	95.8	96.4	96.9	94.7	96.6	91.9	96.9	90.9	97.1	99.2	99
Fa					96.9	96.1	96.6	94.6	96.5	91.8	96.9	90.8	97.1	98.2	98.8
FB						95.5	95.1	94	95.6	91.1	95.7	90.1	95.7	95.8	96.4
hSD-1/2019							98.6	96.3	98.3	91.4	97.8	90.1	96.1	96.4	97
PRV XJ								95.7	98	91.8	97.4	90.4	96.4	96.8	97.5
PRV-GD									96.7	90.8	95.4	88.9	94.4	94.6	95.3
SX1911										92.1	97.2	90.5	96.3	96.6	97.3
Becker											92	93.8	93	91.7	92.3
SC												91.1	97.3	96.9	97.6
Bartha													92.3	90.8	91.3
HLJ-2013														97.1	97.9
GXGG-2016															98.9

**Table 4:** Protein coding variations of JS-2020 strain compared with Ea strain.

Protein Name	Number (aa)	Amino Acid Residues and Location
UL50	1	24 (+E)
UL49.5	1	4 (+S)
UL48	4	R161Q T216M T271A H397P
UL47	7	P399A V404L D406E T411A 414–415(TL > AV) P419A
UL46	2	C494Y A666T
UL27	6	P735L H560Q G393D V114G T112P
UL28	1	V428G
UL30	1	P150L
UL32	1	T402A
UL34	1	A78V
UL35	1	P102S
UL36	34	G3167D 2510–2512(ΔAPP) K2267T 279–280(ΔQS) 942–968(GAAGRAVGGRGGGRGDARAGCARSPTR>ALQAALSAAVAAAVEMLGRLRAQPDE
UL39	1	L571F
UL41	2	S218T Y11H
UL15	2	F545S K169E
UL17	1	D209A
UL10	4	L200H V158A R116Q Q107R
UL9	1	C218R
UL3.5	1	S195P
UL2	2	42–43(ΔGA)
EP0	3	A270G C200S V161M
IE180	7	V999A Q943E N908K P865S V757A T724A Q524R
US1	21	P81A G136C K236E 342–343 (+EDEDEDEDEDEDEDEDED)
US3	5	S24G R115P P135R V177D G214A
US6	2	F169S S278R
US7	1	V148L
US2	1	Y265S

Note: Single amino acid variation is indicated by the amino acid in Ea strain, position and amino acid in JS-2020 strain, e.g., R161Q. Insertions in JS-2020 strain are indicated by position in Ea strain followed by a plus sign and the amino acids of JS-2020 strain, e.g., 24(+E). Deletions are indicated by position, symbol “Δ” and the amino acids in Ea strain, e.g., 279–280(ΔQS). Sequential variations are indicated by position, the amino acid in Ea strain, symbol “>” and the amino acid of JS-2020 strain, e.g., 414–415(TL > AV).

**Table 5:** Amino acid homology of major immunogenic and virulence-related genes.

PRV Strain	Homology of Amino Acid Sequence (%)					
	gB	gC	gD	gE	gI	TK
Bartha	96.5	92.7	97.3	-	-	99.4
CH/GX/PRV/2408/2018	99.3	99.4	99.8	99.3	100.0	100.0
DCD-1	99.2	99.4	99.8	99	100.0	100.0
DL14/08	99.3	99.4	99.8	99.5	100.0	99.7
DX	99.2	99.4	99.8	99.1	99.5	100.0
Ea	99.5	100.0	99.5	100.0	99.7	100.0
Fa	99.7	100.0	100.0	100.0	100.0	100.0
FB	98.3	97.3	99.8	99.1	98.6	99.7
FJ/tiger/2018-1	99.5	99.4	99.8	99.5	100.0	99.7
GXGG-2016	99.7	100.0	100.0	83.9	100.0	-
HeN1	99.3	99.4	99.8	98.8	100.0	100.0
HeNZM/2017	99.3	99.4	99.8	99.3	100.0	100.0
HLJ-2013	97.8	94.4	100.0	99.8	100.0	99.4
HLJ8	99.3	99.4	99.8	99.3	100.0	100.0
HN1201	99.3	99.4	99.8	99.1	100.0	100.0
hSD-1/2019	99.3	99.4	99.8	99.1	100.0	100.0
JS-2012	99.3	99.2	99.8	99.1	100.0	100.0
JS-XJ5	99.3	99.2	99.8	99.3	100.0	100.0
JX/CH/2016	99.2	99.2	99.8	99.1	100.0	100.0
LA	98.8	98.3	99.7	99.5	98.6	100.0
PRV XJ	99.2	99.4	99.8	99.1	100.0	100.0
PRV-GD	99.3	99.4	99.8	99.1	100.0	100.0
PRV-JM	99.3	99.4	99.8	99.1	100.0	100.0
SC	99.7	94.4	100.0	100.0	100.0	99.4
SX1911	99.1	99.4	99.8	99.1	100.0	100.0
TJ	99.3	99.4	99.8	99.3	99.7	100.0
ZJ01	99.2	99.4	99.5	98.4	97.3	100.0

Note: “-” indicates the deletion of this gene.

## Data Availability

All the data generated during this study are included in the manuscript. Additional data related to this article may be requested from the corresponding authors.
